# The mere presence of a smartphone reduces basal attentional performance

**DOI:** 10.1038/s41598-023-36256-4

**Published:** 2023-06-08

**Authors:** Jeanette Skowronek, Andreas Seifert, Sven Lindberg

**Affiliations:** Faculty of Arts and Humanities, Paderborn University, 33098 Paderborn, Germany

**Keywords:** Psychology, Human behaviour

## Abstract

The smartphone has become an indispensable part of everyday life. It enables endless possibilities and offers persistent access to a multiplicity of entertainment, information, and social contacts. The development towards a greater use and a persistent presence of the smartphone does not only lead to advantages, but also raises potential for negative consequences and a negative influence on attention. In this research, the hypothesis of the mere smartphone presence leading to cognitive costs and a lower attention is being tested. The smartphone may use limited cognitive resources and consequently lead to a lower cognitive performance. To investigate this hypothesis, participants aged 20–34 perform a concentration and attention test in the presence and absence of a smartphone. The results of the conducted experiment imply that the mere presence of a smartphone results in lower cognitive performance, which supports the hypothesis of the smartphone presence using limited cognitive resources. The study as well as the subsequent results and the resulting practical implications are presented and discussed in this paper.

## Introduction

The smartphone represents a device that is becoming more and more widespread around the world. According to the Pew Research Center, in 2021 100% of Americans aged 18–29 owned a cellphone of some type, while 96% owned a smartphone^1^. The fact that smartphone usage is increasing is a result of the many advantages it offers. From unlimited communication options to unlimited access to knowledge, the smartphone enables endless possibilities. However, the societal shift towards a greater use of the smartphone raises potential for negative consequences.

People are permanently surrounded by distractions, such as the smartphone. To suppress an impulse like “I want to play on my smartphone now!”, a higher instance must control this impulse to be able to perform. The ability to organize, plan, analyze, and compare past and present actions and to control impulses are described as executive functions^2^. The executive function, as part of a working memory model, was firstly mentioned by Baddeley (1974). In the context of this model, Baddeley proposes that cognitive resources are limited^3^.

In accordance with Baddeley, other scientists assume as well that the working memory is limited in its processing capacity. This has been proven several times^4,5^. The extent to which the cognitive processes are influenced by this is described in John Sweller’s Cognitive Load Theory (1988)^6^. In the Cognitive Load Theory (CLT), the load which arises, for example from new information, is described^7^.

According to the CLT, different tasks may have different requirements as well as different levels of complexity. The intrinsic load of a task can thus be different. If the available working memory resources needed to process the intrinsic and extraneous load are exceeded, the cognitive system cannot process all the necessary information to some extent, which results in poorer cognitive abilities^8^. The extraneous load in a given situation can differ as well. The smartphone could therefore represent a load, namely in the form of an extraneous load.

### Distraction by the smartphone

Smartphones receive notifications, which can trigger noises (sounds) and vibrations. These notifications and potential sounds can be a distraction for humans. Studies show the significant distraction of smartphone notifications, even when participants do not respond to the messages^9^. Another study shows that an involuntary attention system becomes active, which actively listens to the smartphone^10^. It was found that hearing one’s own name activates the same system as the ringing of one's own cell phone or smartphone. Distracting effects of the cell phone could also be shown for college students in the context of a lecture. It was shown that participants who kept their cell phones with them during a lecture performed worse and had a worse recall of the contents of the lecture than participants who did not have their cell phones with them^11^.

According to a review^12^ that particularly focused on the smartphones influence on areas of attention, inhibition, and working memory, strong effects of the smartphone can be seen on precisely these areas. Many studies indicate that the smartphone has negative effects on sleep, stress, and academic performance. Liebherr et al. point to the fact that the smartphone has the potential to negatively affect human cognition. Besides being evident in the context of media multitasking, Liebherr et al. point out that the mere presence of the smartphone can already have effects on cognition^12^.

### Influence on attention by the mere presence and availability of the smartphone

Some of the research claims that even the mere presence or the availability of a smartphone, without having an interaction with it, may already be distracting. Although many of the following studies claim to examine the influence of the effect of the mere smartphone presence, it should be noted that most of these studies only examine the influence of smartphone availability. Smartphone availability describes the continuing ability to receive messages and notifications, while the mere smartphone presence describes a smartphone that is turned off, so that no messages and notifications can be received. In contradiction to that, more recent research does not support the hypothesis that the mere presence of a smartphone influences attention.

First, it was Thornton et al. who found a distracting effect of the smartphone by its mere presence. Students in presence of a turned-on smartphone performed poorly in neuropsychological tests compared to students that did the tasks without the presence of a smartphone^13^. A distracting effect of smartphone presence/availability could also be shown in social interactions. It was shown that the presence/availability of the smartphone forms an interference with the formation of relationships in people by inhibiting the development of interpersonal closeness and familiarity^14^.

Research also shows that the mere smartphone presence and the availability influences working memory capacity and fluid intelligence, which leads to lower attention. Ward et al. shows in a series of studies that smartphone availability depletes cognitive resources and that the consumption of cognitive resources is high even when subjects manage to continue to gather their attention, for example, when they are not looking at their smartphones. In addition to that, results indicate that only the location (desk, pocket/bag, or other room) of the smartphone affected the performance of the subjects. However, there was no effect of smartphone power (smartphone turned off vs. smartphone turned on) on the interference effect of smartphone presence^15^.

Negative effects on cognitive functions due to smartphone availability could also be examined by Ito and Kawahara. The results show that the group of subjects performing a visual-spatial task under smartphone presence takes longer on average to complete the task^16^. Smartphone availability also influences students’ learning and memory. It was shown that subjects without smartphones had a higher recall accuracy compared to those with smartphones. In addition, it was highlighted that the presence/availability of a smartphone and many conscious thoughts of the smartphone impaired memory performance and information retrieval^17^.

Canale et al. investigated whether individual differences in emotion-related impulsivity traits influenced the effect of smartphone availability on cognitive performance. Here, the effect of smartphone availability on cognition could only be detected when the smartphone was turned on. No effect was found when the smartphone was turned off. In both conditions, the smartphone was on the table and the smartphone that was turned on was muted. Therefore, Canale et al. suggest that attentional capacities and visual working memory capacities are associated with incoming messages, or the possibility of receiving them^18^.

Liu et al. tested the relationship between the effect of the phone’s presence on attention and phone activeness, which is defined as the phone’s physical contact state (on desk or held in hand) and the phone’s power state (control, turned on, turned off). Therefore, Liu et al. used a dual-task paradigm, namely the “letter recognition task” and the “luminance-change detection task”. In this way, the influence of smartphone presence on basic perceptual processes was assessed, which builds the precondition for attentional performances. The results suggest that even a minimal awareness of the presence of a phone, including a switched-off smartphone, can cause a distraction^19^.

Recent research also suggests that the effect of smartphone presence or availability on attentional functioning is only present in high-level tasks and not in low-level attentional tasks. Low-level or basal attentional performance describe the ability to recognize and differentiate between stimuli, as well as to select these stimuli and show a reaction to these (e.g. crossing out stimuli, as in the d2-R). High-level tasks involve meta-cognitive decisions, e.g. reacting on changing demands. Thornton et al. did a study with high attentionally demanding tasks and low attentionally demanding tasks. Here, the negative effect of smartphone availability on attentional processes was only found in the context of high-level tasks^13^. Ito and Kawahara corroborate these findings^16^. Canale et al. found that the more demanding a task, the stronger the interference effects on the subjects' cognitive performance^18^.

Schwaiger and Tahir tested the effect of smartphone presence on undergraduate students on several simple and complex attentional tasks as well as on a non-verbal reasoning task. Participants undertook these tests in 4 conditions (phone face up, phone face down, phone in bag or pocket, phone in another room). However, the authors do not define smartphone power (turned-on or turned-off). They found out that the presence had no impact on simple attentional tasks (and fluid non-verbal intelligence), as well as the smartphone presence had no influence on fluid intelligence. However, an effect while solving a more complex attentional task on students was shown: students experienced higher difficulty when their phone was present^20^.

Furthermore, research also states that the effect of smartphone presence and availability on cognition is moderated by several other constructs, such as smartphone dependence and addiction^13,15,17^, Internet use/attachment^16^ or excessive smartphone use^18^. Findings show that strong smartphone users or users that show a tendency to smartphone dependence are more likely to be affected by the effect of the smartphone availability on attentional processes or cognitive functions.

Contrary to that, more recent research does not clearly support that smartphone presence influences attention. For example, Hartmann et al. tested the effect of smartphone presence on different memory functions, namely short-term memory and prospective memory. No overall effect was found for smartphone presence on short-term and prospective memory performance. However, results show that for participants with low smartphone dependency, performance was better when the smartphone was absent than when the smartphone was present. These results show that the effect of smartphone presence that was shown for working memory cannot be generalized for other domains of memory capacity, such as short-term memory and prospective memory^21^.

Ruiz Pardo and Minda did a replication of the study by Ward et al. (2017) and used the same tasks and conditions that Ward et al. used in their second experiment. Ruiz Pardo and Minda could not replicate the findings of Ward et al. and did not find differences between the different smartphone location conditions (on desk, in pocket/bag, outside the testing room), neither on the o-span task, nor on the go/no-go task. Ruiz Pardo and Minda conclude that the presence of one’s phone may not be enough to affect cognitive performance^22^.

### The current research

The present research aims to contribute to the field of smartphone presence leading to lower cognitive functioning. In particular, it will be investigated whether the mere presence of the smartphone affects attention while the smartphone is turned off. This study is conducted to investigate whether the smartphone presence influences the attention of college students. There have been only few studies on the influence of the turned-off smartphone on attention, which is why this work can make an important contribution to the existing research. In addition to the recording of attention performance under the presence of a smartphone, the smartphone dependence of the subjects is also assessed. As smartphone dependence played a crucial role in other research and influenced the effects of the smartphone presence and availability on attention, it should also be considered here.

The current state of research does not clearly show whether the mere presence of a smartphone influences attention. Many circumstances of this effect are still unclear, which is why this study is a contribution to the question of the smartphone being an influence on attention. Furthermore, recent studies, that proved an influence of the smartphone presence on attention, suggest that there is only an effect of smartphone presence and availability on cognitive functions in the context of high-level tasks. On the other hand, it is unclear whether basal skills are affected by the interference effects of smartphone presence. Therefore, the present study examines the possibility that smartphone presence may already have an effect on basal attentional processes. The concentration and attention test used represents a test that examines basal functions and attentional processes. The present paper can give more insights into the circumstances that lead to lower cognitive functioning while the smartphone is present.

It is hypothesized that the mere presence of the smartphone affects attention, which could lead to poorer cognitive performance. The allocation of attentional resources might be divided between the central task and the smartphone. As presented in the Cognitive Load Theory, the smartphone could represent an additional cognitive load in the form of an extraneous load^7^. If attention is used because it is directed to the smartphone and cognitive resources are depleted, resources will be missing from it and performance to complete tasks will be negatively affected.

## Methods

In this experimental study, the assumption is tested whether the mere presence of the smartphone influences attention. College students perform an attention task in or out of smartphone presence. The experiment takes place in form of online video conferences. In addition to the assessment of attention, smartphone dependence is tested, for which the participants fill out a self-reporting questionnaire.

Due to the COVID-19 pandemic and lockdown measures, the methods as well as the target group had to be adapted, which is why the study was conducted via videoconferences with college students.

### Participants

A total of 49 students volunteered to participate in this study. The age ranges from 20 to 34 years. The study takes place in a total of 12 online videoconferences, of which 1–5 people participate in one session at the same time. Data is collected over a three-week period in February and March 2021. The conditions under which the attention test is administered are manipulated by randomly assigning participants to one of the conditions (with or without smartphone). Participants in the smartphone condition are asked to place their smartphone on their desk during the test. The smartphone is switched off and the screen of the smartphone is placed covered on the table, so that the screen is not visible. The other condition is the without smartphone condition, in which the participants switch off their smartphone and place it outside the room. Seven subjects are excluded from the study. Reasons included completing the test incorrectly, not having test materials that work or participants indicating that they had already known the attention test in advance, which would lead to biased results. Thus, the final sample consists of 42 participants (45.2% females, *M*_age_ = 27.29, SD_age_ = 2.87). 21 results are from participants in the without smartphone condition, whereas 21 results are from participants in the group who performed the attention test in the presence of a smartphone. Participants were informed about the relevant procedure of the study. Informed consent was obtained from all the participants. Subjects’ participation in the study was voluntary and confidential, and participants obtained no compensation. The study was approved by the audit committee of the Faculty of Arts and Humanities of Paderborn University according to the guidelines of the DGPS (*Deutsche Gesellschaft für Psychologie—*German Society for Psychology). All methods used in the experiments performed during the present study were performed in accordance with the relevant guidelines and regulations of the research ethics committee of Paderborn University.

### Measures

#### Attention

Attention is assessed by using the *d2-R concentration and attention test* (*d2-R*)^23^. The d2-R is a strikethrough test in which objects and characters must be searched for that are located between similar objects and characters. The task is to mark the correct ones among many characters. The characters are the letters ‘d’ or ‘p’ and one to four marks, which can be below or above the letter. Participants had to cross out the ds with two marks. The d2-R test consists of a sheet on which are 789 characters. The total time to complete the test is 4 min and 40 s. The test consists of a total of 14 lines and participants have 20 s of processing time per line. If a participant completes the test without errors, 359 characters would be crossed out.

The d2-R can be seen as a test consisting of tasks that are cognitively not very demanding but can be completed quickly^24^. Differences in the ability to concentrate become apparent by the speed and accuracy with which large numbers of such non-demanding tasks can be completed continuously. The d2-R was chosen as the test for this study because it is one of the most widely used methods for measuring attention in German-speaking countries. It is a validated instrument and proven as reliable and dependable^23^.

The d2-R provides information about the processing speed, accuracy and attention performance of the test subjects. The attention performance score, referred to as the AP score, indicates a person's ability to focus their attention^23^. The AP score is a measure of processing speed that was adjusted for made errors. In addition to the attention performance score, the speed score, called PTO score (*processed target objects*) and the E% score are assessed. The standardized E% score indicates the accuracy of a test person in processing the d2-R^23^.

The speed, the accuracy as well as the attention performance are recorded by the number of processed target objects (i.e. crossed out target objects) as well as the errors made, i.e. crossing out distractors or not crossing out target objects^23^.

#### Smartphone dependence

Smartphone dependence is assessed by using the *German short version of the smartphone addiction scale* (*d-KV-SSS*). This scale was selected to prioritize instruments that have been validated^25^ and proven as a reliable and dependable instrument in a German speaking region ^26^. Montag (2018) adapted the original scale (*short version of the smartphone addiction scale*) by Kwon et al. (2015)^25^ via a traditional translation and back-translation procedure and developed the German short version of the Smartphone Addiction Scale (d-KV-SSS)^26^. Modifications of the d-KV-SSS were made in the present study to increase comprehensibility, as well as to make it more personal to the participants (see supplementary information for the questionnaire). For example, educational language words as well as sentence constructions were simplified and adapted.

The d-KV-SSS questionnaire can reveal a possible smartphone dependence as well as tendencies towards a smartphone dependence. The items used in the questionnaire can be answered using a six-point Likert scale. Participants can give answers ranging from "I do not agree at all" (1) to "I fully agree" (6). These responses to the 10 items are summed to obtain an overall score of smartphone dependence. The total score thus ranges from 10 to 60 points. Higher scores are associated with a stronger tendency towards smartphone dependence^26^. Cronbach’s Alpha was 0.78.

### Procedure

The experiment is conducted in form of digital video conferences via a video platform. In advance, participants receive detailed instructions on how to prepare for the test, which include a detailed description and a sketch of how they should position themselves and organize their surroundings during the test (see supplementary information for the handed-out instructions and the sketch). Participants are advised to sit at an empty desk with only a pen, their smartphone and the envelope sent to them, which contains the test materials. In addition, participants are told to eliminate all possible distractions. Furthermore, participants receive a note to also inform possible family members, roommates, etc. that they must not be disturbed during the experiment. It is emphasized as particularly important that all functions and notifications of the participants’ own laptop or computer, except for the function of the video call, are disabled. Participants receive a detailed list of what to switch off. This is to ensure that the laptop or computer serves only as a camera and audio source and is not a distraction.

Besides the instructions, participants receive the d2-R test and the questionnaire d-KV-SSS by mail. Whether the instructions are implemented can be observed during the video call by the supervisor. If the instructions are not implemented correctly or sufficiently, participants will be informed by the supervisor. This ensures that the instructions are implemented correctly.

The experiment is conducted in a session over a period of approximately 20 min. Since the study takes place via an online videoconference, participants are in their usual environment and a representative situation is given. The situation created in the experiment, in which the participants are at their desk, working on a task and having their smartphone next to them, resembles an everyday life situation and can be transferred to it.

Before the testing, precise instructions are prepared. To keep confounding variables as low as possible, a video is sent to the participants in advance in which the experimenter gives instructions for the attention test (d2-R). Participants are advised to not watch the video until the beginning of the videoconference. The instructions in the video include a reminder of the instructions that should be carried out before the experiment and a brief description of the course of the test.

In the further course of the conduction of the d2-R, the instructions are read aloud by the experimenter. The experimenter adheres to the exact, prepared wording to create the most equal conditions possible for all participants. When the conduction of the d2-R begins, the instructions given in the test manual of the d2-R are used.

Besides the advantage of the video, that the instructions are the same for all participants, there is another positive aspect. The participants are instructed to download the video to their smartphone in advance. The video, which the subjects watch on their smartphones during the testing, ensures that all subjects own a smartphone. The subjects can be instructed immediately after watching the video to place their smartphone in a specific location without receiving a hint about the experimental manipulations as well as the hypotheses to conduct a blinded experiment. Participants are given the information that the general development of college students’ attention is examined.

After the participants have watched the video, they are advised where to place their smartphone. Depending on the group they are assigned to, the smartphone is either placed on the table or taken to another room. Subsequently, the participants create an individual personal code to ensure that completed documents can be assigned to each other, but at the same time the results remain anonymous. Next, the d2-R is conducted. During the test conduction, after every 20 s, the experimenter gives the cue "Stop. Next line." until after 4 min and 40 s the cue "Stop. Put the pen aside." is given and the test is completed. After completing the d2-R, participants answer the d-KV-SSS to gather information about a possible tendency towards smartphone addiction.

## Results

### Statistical analyses

To test the hypothesis that subjects have lower attention performance when the smartphone is present, the d2-R concentration and attention test is conducted. The performance score of the d2-R represent the individual attention performance scores. The performance is evaluated for both conditions (with or without the smartphone). In addition, the German short version of the smartphone addiction scale is evaluated. Stronger and weaker tendencies towards smartphone addiction are examined. The results of the d2-R and the questionnaire are evaluated using SPSS Statistics for Mac. An effect could be shown.

A one-factor ANOVA was used to examine the subjects’ attention performance in connection with a tendency towards smartphone addiction. The presence or absence of the smartphone is the independent variable, and the attention performance score is the dependent variable. Group comparisons were made using the ANOVA. Additionally, a covariance analysis was conducted to picture the potential moderating effect of the covariate “smartphone addiction”.

### Effects of smartphone presence on attention

For the hypothesis that people have lower attention performance when the smartphone is present, three one-tailed ANOVAs are performed with the presence or absence of the smartphone as the independent variable and one of the three given scores of the d2-R (attention performance, speed and error-value) as the dependent variable.

To perform an ANOVA, the following necessary assumptions for the validity of the results of an ANOVA are checked: the normal distribution of the scores, the homogeneity of the variance and the independence of the samples. There was no normal distribution given for all values. As an ANOVA is a robust test, an ANOVA was chosen as the used test. We use an alpha level of 0.05 for all statistical tests.

To test the hypothesis that there is a significant difference between the two groups, an ANOVA is performed with attention performance score (AP score) as the dependent variable and condition (with or without smartphone) as the independent variable. In addition, the effect sizes are given and are classified into small (η^2^ = 0.01), medium (η^2^ = 0.06), and large (η^2^ = 0.14) effects. For the classification of the effect size η^2^, reference values for classification according to Cohen^27^ are used.

The means of the AP, PTO and E% scores for the two conditions (with and without smartphone) are shown in Table 1. A significant effect was found (*F*(1,40) = 6.168, *p* = 0.017, η^2^ = 0.134). The without smartphone group has significantly higher attention performance (*M* = 108.95) compared to the with smartphone group (*M* = 99.71). There is a tendency towards a large effect. In addition to the AP score as a dependent variable, the ANOVA is performed with the speed score (PTO score) as dependent variable and the condition (with or without smartphone) as independent variable. A significant effect is also found for this ANOVA (*F*(1,40) = 7.592, *p* = 0.009, η^2^ = 0.160). The significance of this ANOVA means that the subjects under the smartphone condition (*M* = 98.48) have a significantly lower work speed compared to the without smartphone condition (*M* = 108.57). A large effect is present here.

**Table 1:** Descriptive statistics of the results of the d2-R test.

Group	Factor	N	Mean	SD
Without smartphone	AP	21	108.95*	14,538
	PTO	21	108.57**	12,975
	E%	21	102.81	6743
With smartphone	AP	21	99.71	8900
	PTO	21	98.48	10,657
	E%	21	105.91	7134

*AP* attention performance, *PTO* processed target objects, *E%* error value, * = *p* < .05, ** = *p* < .01.

To test the hypothesis that there is a difference between the without smartphone and with smartphone groups for accuracy (E% value), an ANOVA is performed with the E% score as the dependent variable and the condition (with or without smartphone) as the independent variable (*F*(1,40) = 2.088, *p* = 0.156, η^2^ = 0.050). The result of this ANOVA is not significant, meaning that the subjects do not have significantly different scores in accuracy under the two conditions.

Thus, the ANOVA revealed that participants under the smartphone presence condition show significantly lower performance on the d2-R attention test compared to participants who complete the attention test in the absence of the smartphone. Both attention performance scores (AP scores) and working speed (PTO scores) are significantly lower in the smartphone presence group compared to the smartphone absence group. Only accuracy (E% score) does not differ significantly.

### Moderation by smartphone dependence

The mean for the d-KV-SSS for the entire group is *M* = 29.47, *SD* = 8.38. The mean for the group of subjects in the with smartphone condition is *M* = 29.80, *SD* = 8.89. For the group in the without smartphone condition, the mean is *M* = 29.14, *SD* = 8.04. No significant mean difference is found, *F*(40,1) = 0.065, *p* > 0.05.

Thus, overall, no strong tendencies toward smartphone dependence can be found for the entire group of subjects. If the mean of the total group is considered, it is *M* = 29.47 and thus forms the midfield. This shows that there are no strong, but also no low tendencies towards smartphone dependence.

The possibility of smartphone dependence as a moderating variable is examined. For this purpose, covariance analysis is performed with the variable of smartphone presence or absence. This analysis shows no significant interaction of the variables. Thus, a moderation of the effect of the presence of the smartphone by the individual differences in the dependence on the own smartphone cannot be confirmed.

## Discussion

Students are increasingly using the smartphone^28^ and smartphones are permanent companions to people worldwide. As a result, they have the potential to significantly influence consumers.

Through the present paper, the possibility of a strong negative influence by the smartphone is investigated. Especially college students can be negatively influenced by the heavy use of the smartphone. The work presented here shows one consequence of the smartphone’s integration into the everyday life of students: the loss of attention. It provides evidence that even the mere presence of one’s smartphone consumes cognitive resources, without willingly shifting attention or actively using the smartphone. In the test situation, there was no visible interaction with the smartphone, as the smartphone was not looked at or picked up. This work was able to show in more detail to what extent attention performance is negatively affected: it was found that people show a slower work speed in the presence of a smartphone. New findings were obtained regarding the influence of smartphone presence on attention in the context of complex and non-complex tasks. Contrary to the previous state of research, a strong influence of smartphone presence was found in the context of tasks that require basal attentional processes, which is a new finding in this area. Since previous research cannot clearly show whether the mere presence of a smartphone influences attention, the results of this study represent a contribution to this and support the assumption that smartphone presence is a negative influence on attention.

The theoretical framework presented here proposes the consumption of cognitive resources as an explanation for the different performances. As the theoretical presentation of the executive functions show, the cognitive resources of humans are limited^3–6^. Thus, the limited resources must be divided. According to the Cognitive Load Theory, resources are consumed by different types and amounts of cognitive loads, which consequently leads to different cognitive performances^7^. The fact that attention was visible from the outside, but the subjects' performance was nevertheless lower in the presence of the smartphone, provides evidence of an invisible consumption of cognitive resources. According to Sweller’s categories for the classification of loads^7^, the smartphone presence can represent an extraneous load, as it is independent of the task and the content to be processed. The additional load represented by the smartphone consumes cognitive resources that are consequently no longer available to process further information. The additional load of the smartphone can thus be used to identify the reason for the negative influence of the smartphone on attention and consequently the lower performance under smartphone presence.

Other studies have already shown that attention and performance decreases when a task is performed while the smartphone was available, which means that there was still the possibility to receive messages and notifications. The present study provides evidence that even the mere presence of one’s smartphone consumes cognitive resources, without any voluntary shift of attention or active use of the smartphone. The present findings are consistent with previous research findings that showed a reduction of attention and performance due to the presence/availability of the smartphone^13,15–18,20^. Additionally, Ito and Kawahara found that subjects show slower performance when a smartphone is available^16^. The present work can corroborate and extent the findings of Ito and Kawahara, as it could be shown that even the mere presence of the smartphone has the discovered effect of cognitive interference and lead to a slower work pace.

Moreover, Koessmeier and Büttner tested the mere presence effect of the smartphone while also tracking visual distraction that might be caused by smartphone presence. Participants wore mobile eye tracking glasses while performing cognitive tasks and reading tasks in smartphone presence and absence. Negative effects of smartphone presence on performance could not be replicated. During the task, participants almost never looked at their smartphone. Only during breaks and transitions between tasks, people drew their visual attention to their smartphones. Results show that the presence of the smartphone increased smartphone vigilance, however, this did not influence task performance. Koessmeier and Büttner conclude that people can regulate the visual attention that they pay to smartphones^29^.

The effect of smartphone presence and availability, i.e. the loss of attention, was only found for complex or high-level tasks in the prior body of research^13,16,18,20^. The results of our study are opposite to this. The d2-R represents a simple test with little complexity, where the subjects must discriminate simple visual stimuli and which consists of intellectually simple tasks^23^. Nevertheless, the presence of the smartphone had the effect of reducing attention performance. Moreover, a strong effect was shown, even in the context of a task that requires basal attentional processes. Thus, our study contributes to the previous findings regarding the effect in connection with tasks requiring basal or high-level attentional performance. It is shown that the smartphone presence has a negative effect on attention, even while solving non-complex tasks.

A strong difference was found to Canale et al. who found in their studies that there was only an effect for the presence of the smartphone when it was switched on and not when it was switched off^18^. Results of Ward et al. (2017) and Thornton et al. show that a turned off smartphone influences attention^13^. Liu et al.^19^ also found a possible effect that having a turned-off smartphone present reduces attention. In our study, effects were found for a smartphone that was switched off. It could be shown that cognitive capacities are used by the mere presence of a smartphone even if there is no possibility to receive notifications. These results imply that the complete spatial separation from the own smartphone is a possibility to counteract a negative influence of the smartphone on the ability to focus attention. This also corresponds to the results of Ward et al., who stated as well that only the spatial separation counteracts a negative influence of the smartphone on attention^15^.

In contrast to the negative effects presented here, positive effects are also possible due to smartphone presence. In another study, Liu et al. investigated whether the presence of a smartphone leads to higher levels of creativity. Previous research shows that environmental factors can change a state of attention into a diffused state, which fosters creativity. Results of Liu et al.’s study show that creativity increases when a smartphone is present, as workers' attentional state is affected by the presence of the smartphone. Due to the presence of the smartphone, workers are in a diffused state of attention, which leads to a better performance on a creativity task^30^. Thus, the study shows possible positive influences due to smartphone presence, which provides further indications for a positive handling of the smartphone: These results show that in certain contexts, the presence of a smartphone can have positive influences.

An influence of smartphone dependence on the effect of smartphone presence could neither be confirmed nor refuted here, as no strong smartphone dependence tendencies were present in the subjects. Given the recent increase in smartphone use, smartphone dependence was considered to be a relevant factor, and because smartphone dependence has played an important role in previous studies, this factor was controlled for. As smartphone addiction is not that common^1^, these findings are consistent with the overall prevalence. However, as previous research by Ward et al.^15^, Thornton et al.^13^, and Ito and Kawahara^16^ found an effect of smartphone dependence and personal relevance of the smartphone, this should be further investigated in future research.

### Limitations

By conducting the attention and concentration tests via an online video platform, limitations for the given results should be considered. Limitations arise from the fact that the general test situation during the examination was no laboratory situation, which means that various influencing factors could be present. Possible external factors may have interfered with the participants attention. However, this limitation is also contrasted with the advantages of a situation in which the subjects are in their natural environment. Salvucci and Taatgen (2011) did a study on multitasking, in which they argue that a laboratory experiment cannot represent the true costs of multitasking, as experiments in the laboratory are not representative for multitasking situations in real life situations^31^. Thus, an influence on cognitive abilities can also be given by the laboratory situation compared to an everyday life situation, which is why keeping the participants in their natural environment is also an advantage given in this study. To counteract the different conditions that were given for the participants, exact instructions were prepared in advance and used while conducting the experiment. However, differences in instruction cannot be ruled out. Furthermore, participants in our study had their personal smartphones with them. Thus, each participant had a different smartphone, which also led to different conditions and the possibility of different influences on the participants. However, Ward et al. showed that the effect of smartphone presence on attention is mostly triggered by the personal smartphone, as the personal relevance of this device plays an important role^15^. As mentioned by Liu et al., the influence of cellphone ownership on the effect of smartphone presence was not examined enough in the currently existing research and should be further considered in future studies^19^.

Limitations also result from the sample of our study. First, with 42 test persons, only a relatively small sample was given. Second, the sample used in the present research was predominately White (93%), highly educated, and relatively young (20–34 years). Thus, it is not clear if the present results can be generalized to the broader population using a smartphone. To investigate the generalizability of the present findings, more studies on the effect of smartphone presence should be done, especially concerning a more diverse sample group.

### Practical implications

As our data suggests that the mere presence of the smartphone has a negative effect on attention, implications for the use of smartphones in students’ daily lives can be drawn. In general, students should be made aware of the fact that the presence of the smartphone negatively affects attention. This can be done, for example, at universities in form of posters. Based on that, students should change the way they use their smartphone: they should not carry it with them permanently, and students should make sure that they do not have their smartphones in their field of vision, especially while studying or attending a lecture or class. Furthermore, the general rules and limitations for smartphone use both at universities and in other learning contexts, such as schools, should be questioned based on the present findings. In addition, the question can be raised how much other objects of “mobile technology”, such as tablets, have the potential to negatively influence cognitive performance. In the course of digitalization, many efforts are being made to implement digital devices as much as possible in the classroom and everyday teaching, especially now in the context of the COVID-19 pandemic^32^. However, as results of this study show, placing the smartphone in another room is already sufficient to counteract the resource-consuming effect of the smartphone. This can be implemented by students in different contexts, for example while studying, to counteract the consumption of cognitive resources. In this way, better cognitive performance can be achieved by students.

### Conclusion

The hypothesis that the mere presence of the smartphone influences attention without any interaction with it can thus be confirmed by this study. It could be shown that the presence of the smartphone results in lower attentional performance. In addition, with the help of this work, the type of influence on attention by the smartphone can be shown more specifically: the presence of the smartphone has a negative influence on the working speed and thus on cognitive performance and attention. Under the presence of the smartphone, individuals work slower.

Taken together, most studies show an influence of a turned-on or available smartphone on attention in the context of high-level attentional tasks. This study shows an influence of the mere presence of the smartphone in the context of low-level attentional performance, which is a contribution to the previous body of research.

The theoretical framework shows that the smartphone represents an additional cognitive load, whereby limited cognitive resources are consumed. As a result, cognitive performance is negatively affected. The results of this paper suggest that especially the speed of cognitive performance and processing is impaired. In addition, an important fact could be shown by this study: the smartphone presence influences attention even while solving tasks that require basal attentional processes.

Based on these results, the everyday use of smartphones and the desire for a greater digitalization, and thus the increased implementation of digital devices in our daily lives, should be critically reflected. With this work, concrete possibilities for a positive handling of the smartphone can be given. It could be shown that it is not sufficient to cover the screen of the smartphone or to turn it off. However, placing the smartphone in a different room is sufficient to avoid the negative effects on attention performance. This is a simple and effective method to achieve this.

As the results of this study show, the use and permanent presence of a smartphone imposes a great challenge on society. At the same time, current studies show that the smartphone and also the smartphone presence can have positive effects. For example, during the COVID-19 pandemic, the smartphone proved to be a particularly useful tool. All of this shows that in order to get the most out of a smartphone and make the best of its costs and benefits, a balanced and conscious use of the smartphone must be achieved.

Thus, it can be shown that smartphone use should be critically scrutinized and should only be done in appropriate contexts. Students should avoid having their smartphone with them while attention is required. Based on this work, many other contexts can be explored in which the smartphone could be a negative influence on attention.

The possibilities of the smartphone are enormous, however, this work shows that the advantages of the smartphone come with a strong negative disadvantage: high cognitive costs and strong losses of cognitive abilities.

## Supplementary Information

Supplementary Information.

## Data Availability

Data is available on zenodo.org: https://doi.org/10.5281/zenodo.7014618.
