# Supplementary material for: The mere presence of a smartphone reduces basal attentional performance
Source: Sci Rep. 2023 Jun 8;13:9363. doi: 10.1038/s41598-023-36256-4 (PMC10249922; doi:10.1038/s41598-023-36256-4)
Supplement: Supplementary file 1 — Supplementary Information. [file 41598_2023_36256_MOESM1_ESM.pdf]

The mere presence of a smartphone reduces basal attentional performance

Jeanette Skowronek[1*], Dr. Andreas Seifert[1], Prof. Dr. Sven Lindberg[1]

[1]Paderborn University, Faculty of Arts and Humanities, Paderborn, 33098, Germany
[*]jeanette.skowronek@uni-paderborn.de

Supplementary Information 1: Previous Instruction

Information handed out to the participants before the conduction of the experiment

Thank you for participating in my study!
Our study is about the attention performance of college students. You will take an attention test and fill out a short questionnaire.

Your ability to focus your attention will be examined. For this it is important that possible distractions are eliminated beforehand so that your test results are not falsified.
You can already make these preparations before the *Zoom* meeting.

To do this, you should note the following points:

- The *Zoom* meeting must be run on a PC or laptop (NOT on a smartphone).
- If possible, please install *Zoom* on your computer and do not open it via the browser.
- It is also important that all possible distractions (such as your landline phone, or any other source of noise) are turned off.
- If you have family members, roommates, etc. living with you, inform them before the experiment that they are not allowed to disturb you during the experiment (if needed, you have also been sent a "Do Not Disturb" sign that you can stick on the door of your room).
- It is also very important that all notifications on your laptop are turned off.
- Check if these features of your laptop are turned off (please check and tick off if necessary):
  - All your messengers (like *WhatsApp*, Telegram, Signal, Slack, Skype etc.)
  - E-Mail accounts
  - Push messages from your browser (especially important if the *Zoom* app is not installed and *Zoom* is opened from your browser)
  - Push messages from other programs (like antivirus programs)
  - Push messages from your operating system (e.g. Windows or macOS. You can turn this off under "Settings" and "Notifications")

- Position yourself as shown on the attached sketch, i.e.:
  - You are sitting at an empty desk
  - In front of you is only the envelope I have sent you and a pen (if possible a ballpoint pen).
  - Your laptop/ PC must be placed BEHIND you.

- Your laptop/ PC should have a distance of about 1,5 meters to you.
- At the same time, you must still be able to hear my instructions well over *Zoom*

You will receive a video from me with further instructions. You will watch this video on your smartphone during the *Zoom* meeting. Download this video to your smartphone before the *Zoom* meeting. You will receive all further information during the *Zoom* meeting.

Sketch of the positioning during the attention test

Herewith you get a sketch on which you can see how you should position yourself and your laptop during the test. Implement the sketch (as best you can). You will also be given a "Do not disturb" sign to stick on your room door if needed.

Figure S1. Position during the attention test: sketch

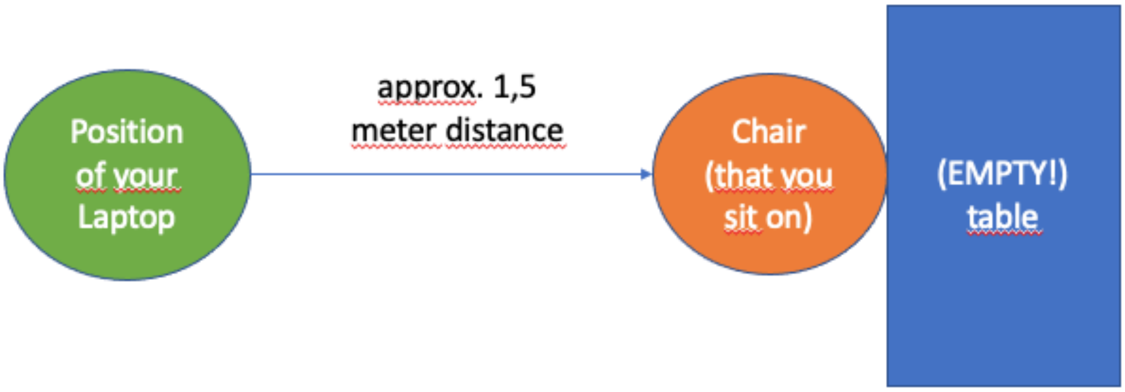

Supplementary Information 2: Questionnaire

The *Smartphone Addiction Scale* was developed by Kwon, Kim, Cho, and Yang (2013)[2]. Based on this questionnaire, the *Short Version of the Smartphone Addiction Scale* was developed by Kwon et al. (2013)[2]. Montag (2018) adopted this questionnaire and developed the *German Short Version of the Smartphone Addiction Scale* (*d-KV-SSS*)[1]. This version by Montag (2018), slightly modified, is used in the present study. Montag (2018) translated and slightly modified the questionnaire by Kwon et al. (2013), resulting in the d-KV-SSS. The accuracy of the translation was verified by Montag (2018) by back translating the questionnaire from German into English by a second person[1].

Further modifications of the d-KV-SSS were made in this study to increase comprehensibility, as well as to seem more personal to participants. Educational language words as well as sentence constructions were adapted and simplified.

An example of the change and simplification of words can be found in the first question. Montag's original questionnaire states, "I miss completing planned tasks due to my smartphone use"[1]. This item was changed to "I miss doing planned tasks because of my smartphone use".

In addition, only the aspects that were relevant to the college students participating in the experiment were filtered out. For example, item two "It is difficult for me to concentrate because of my smartphone use in school, study, or during work" was changed to "It is difficult for me to concentrate because of my smartphone use in study or during work," since it can be assumed that the college students are no longer concerned with school.

The questionnaire is in the form of a 6-point Likert scale and there are 6 items to be answered. The scale ranges from "I do not agree at all" (1) to "I strongly agree" (6). By scaling the answers from 1 to 6 for 10 questions, a total score between 10 and 60 points can be obtained. This value can then be evaluated as an existing or non-existing tendency to a possible smartphone addiction[2].

This is the final questionnaire used in our study to assess smartphone dependence.

1. Ich verpasse es, geplante Aufgaben zu erledigen, weil ich mein Handy nutze	1	2	3	4	5	6
2. Es fällt mir schwer, mich aufgrund meiner Handy-Nutzung auf der Arbeit oder in der Universität zu konzentrieren	1	2	3	4	5	6
3. Ich empfinde Schmerzen in Handgelenk oder Nacken während ich mein Handy benutze	1	2	3	4	5	6
4. Für mich wäre es nicht auszuhalten, kein Handy zu besitzen	1	2	3	4	5	6
5. Ich fühle mich ungeduldig und unruhig, wenn ich mein Handy nicht bei mir habe	1	2	3	4	5	6
6. Ich denke sogar dann an mein Handy, wenn ich es nicht nutze	1	2	3	4	5	6
7. Ich werde niemals aufhören mein Handy zu nutzen, selbst dann nicht, wenn mein Alltag sehr stark davon beeinflusst ist	1	2	3	4	5	6
8. Ich überprüfe mein Handy andauernd, um keinen Chat mit anderen in Apps wie Facebook, Instagram, Snapchat oder *WhatsApp* zu verpassen	1	2	3	4	5	6
9. Ich nutze mein Handy länger als beabsichtigt	1	2	3	4	5	6
10. Die Menschen um mich herum sagen mir, dass ich mein Handy zu stark nutze	1	2	3	4	5	6

Table S1. Modified questionnaire based on the *German Short Version of the Smartphone Addiction Scale* (*d-KV-SSS*) by Montag (2018)[1]

This is the questionnaire translated to English. However, we only used the German version in our study.

1. I miss doing scheduled tasks because I use my smartphone	1	2	3	4	5	6
2. It is difficult for me to concentrate at work or at university because of my cell phone use	1	2	3	4	5	6
3. I feel pain in my wrist or neck while using my smartphone	1	2	3	4	5	6
4. it would be unbearable for me not to have a smartphone	1	2	3	4	5	6
5. I feel impatient and restless when I do not have my smartphone with me	1	2	3	4	5	6
6. I think about my cell phone even when I am not using it	1	2	3	4	5	6
7. I will never stop using my smartphone, even if my daily life is very much influenced by it	1	2	3	4	5	6
8. I constantly check my phone, so I don't miss a chat with others on apps like Facebook, Instagram, Snapchat or *WhatsApp*	1	2	3	4	5	6
9. I use my phone longer than intended	1	2	3	4	5	6
10. People around me tell me that I use my smartphone too much	1	2	3	4	5	6

Supplementary Information 3: Results of the *German Short Version of the Smartphone Addiction Scale* (*d-KV-SSS*)

	Question 1	Question 2	Question 3	Question 4	Question 5	Question 6	Question 7	Question 8	Question 9	Question 10
Mean	2.88	2.88	2.29	3.60	2.98	2.31	2.52	3.14	4.43	2.45
Std.-Deviation	1.234	1.565	1.453	1.639	1.585	1.199	1.366	1.507	1.382	1.418
Variance	1.522	2.449	2.111	2.686	2.512	1.438	1.865	2.272	1.909	2.010

Table S2. Results of the Questionnaire *d-KV-SSS*

References

1. Montag, C. *Homo Digitalis. Smartphones, soziale Netzwerke und das Gehirn* (Springer, 2018).

2. Kwon, M., Kim, D., Cho, H. & Yang, S. The Smartphone Addiction Scale: Development and Validation of a Short Version for Adolescents. *PLoS One* **8**, e83558; 10.1371/journal.pone.0083558 (2013).
